# Remote Sensing and Geographic Information Systems in Epidemiology

**DOI:** 10.3201/eid0804.010529

**Published:** 2002-04

**Authors:** Uriel Kitron

**Affiliations:** University of Illinois, Urbana, Illinois, USA

The applications of remote sensing and geographic information systems (GIS) to vector-borne and helminthic diseases have progressed far beyond the pretty pictures which dominated their early use. As Wood et al. indicate in the last chapter, the number of papers in the area has increased drastically over the last decade, in number and sophistication. The editors of this special volume of Advances in Parasitology have been in the forefront of applying statistical and biological approaches to the mapping of vector-borne diseases and have brought together experts to review existing knowledge, identify gaps in understanding, and describe future applications of these powerful approaches. 

This book is a timely overview of satellite imagery, GIS, and spatial statistics. The emphasis is on vector-borne diseases, with one chapter devoted to helminthic diseases. With the exception of the chapter on spatial statistic and GIS, there is little mention of other epidemiologic applications (e.g., GIS and cancer, AIDS, and environmental health). The book is divided into three parts: three introductory chapters describing the methodology; four chapters which review the applications and provide examples from the authors’ experiences in studying African trypanosomiases, malaria, tick-borne diseases, and human helminthic diseases; and three concluding chapters which describe environmental variables, disease risk forecasting, and the education about and future of remote sensing in human health.

Although remote sensing, GIS, and spatial statistics have been reviewed separately elsewhere, the encompassing review, the inclusion of lists of URLs, and the extensive references make the introductory chapters timely and instructive for new users. The audiences that will benefit most from the book include researchers and public health administrators who want to integrate these tools into research, surveillance, and control efforts. This audience, as well as more experienced users, can gain much from the chapters that provide examples of specific applications derived from deep understanding of the biology of disease. The chapters by Rogers, Randolph, and Brooker and Michael, in particular, are based their own research and expertise in trypanosomiasis, tick-borne diseases, and helminthic diseases, respectively.

Remote sensing and GIS are particularly relevant to emerging infectious diseases. The chapter entitled Forecasting Disease Risk for Epidemic Preparedness provides a road map for developing early warning systems. While this chapter, like the rest of the book, is clearly written by advocates of the applications of remote sensing and GIS, the authors remain aware of critical issues, such as the distinction between statistical and biological models and the notion that insights gained by false negatives and positives predicted by models are as important as successful predictions. Other issues that have hampered more extensive applications of remote sensing and GIS to vector-borne diseases include lack of training, gaps in data (quality and quantity, particularly of epidemiologic and parasitologic data), inadequate tools for data gathering, and limits on management and understanding. This book goes a long way to address these issues and is likely to lead to more and improved applications of remote sensing and GIS.

